# Knee laxity, joint hypermobility, femoral anteversion, hamstring extensibility and navicular drop as risk factors for non‐contact anterior cruciate ligament injury in female athletes: A 4.5‐year prospective cohort study

**DOI:** 10.1002/ksa.12625

**Published:** 2025-02-13

**Authors:** Kati Pasanen, Arttu Seppänen, Mari Leppänen, Kari Tokola, Timo Järvelä, Tommi Vasankari, Grethe Myklebust, Tron Krosshaug, Jari Parkkari

**Affiliations:** ^1^ Integrative Neuromuscular Sport Performance Laboratory, Faculty of Kinesiology University of Calgary Calgary Alberta Canada; ^2^ Sport Injury Prevention Research Centre, Faculty of Kinesiology University of Calgary Calgary Alberta Canada; ^3^ Tampere Research Center of Sports Medicine, UKK Institute Tampere Finland; ^4^ Faculty of Medicine and Health Technology Tampere University Tampere Finland; ^5^ Tampere University Hospital Tampere Finland; ^6^ UKK Institute Tampere Finland; ^7^ Sports Medicine and Arthroscopic Center, Hospital Mehiläinen Tampere Finland; ^8^ Department of Sports Medicine, Norwegian School of Sport Sciences Oslo Sports Trauma Research Center Oslo Norway

**Keywords:** basketball, female athlete, floorball, ice hockey, injury risk, volleyball

## Abstract

**Purpose:**

To investigate whether six selected anatomical variables were associated with non‐contact anterior cruciate ligament (ACL) injury in female team sport athletes.

**Methods:**

Two hundred eighty‐seven female athletes (age 13–38 at baseline) from basketball, floorball, ice hockey and volleyball completed a baseline physical examination, including measurements of anterior‐posterior (AP) knee laxity, knee hyperextension, generalized joint hypermobility, femoral anteversion, hamstring extensibility and navicular drop. Athletes entered the study either in 2011, 2012 or 2013 and were followed up until the end of 2015. During the follow‐up, all complete and magnetic resonance‐verified ACL injuries were recorded.

**Results:**

Twenty‐three non‐contact ACL injuries were recorded. There were no significant differences in baseline physical examination variables between athletes who sustained ACL injuries and those who did not. However, a side‐to‐side difference in AP knee laxity greater than 2 mm was observed in 20% of the ACL injury group compared to 12% of the non‐injured group, although this difference was not statistically significant.

**Conclusions:**

In this study, AP knee laxity, knee hyperextension, generalized joint hypermobility, femoral anteversion, hamstring extensibility and navicular drop were not associated with increased risk for non‐contact ACL injury in female team sport athletes. This study was powered to detect moderate to strong risk associations; thus, smaller risk associations may not have been identified.

**Level of Evidence:**

Level II.

**Abbreviations**
ACL: anterior cruciate ligament
AP: anterior‐posterior
BMI: body mass index
KT‐1000: Knee Laxity Testing Device 1000
*p*‐Value: probability value
SD: standard deviation
SPSS: Statistical Product and Service Solutions
SSD: side‐to‐side difference

## INTRODUCTION

Anterior cruciate ligament (ACL) injury of the knee is a serious problem in team sports, which involve repetitive cutting and pivoting movements with high demands on the knee joint [1, 21, 37]. ACL injuries are associated with large healthcare costs, time loss from sports and reduced physical activity, in addition to a significant risk for osteoarthritis, severe pain and disability [28, 32, 45, 51]. This injury affects females more often than males, and it has been estimated that female athletes have a four to six times higher risk of tearing the ACL than their male counterparts [17].

Our understanding of ACL injury mechanisms has evolved significantly over the years, with numerous studies shedding light on various biomechanical factors [24, 25, 27, 34], such as knee valgus [25] and anterior tibial translation which typically occur at the time of injury [24]. The majority of ACL injuries occur in non‐contact situations during sports activities that involve sudden direction changes, decelerations, and jump landings [10, 17, 30, 35, 36]. As knowledge of injury mechanisms has improved, the focus has shifted towards identifying intrinsic factors that may lead to poor knee biomechanics. Several anatomical factors, such as anterior‐posterior (AP) knee laxity, knee hyperextension, generalized joint hypermobility, femoral anteversion, hamstring extensibility and navicular drop, have been proposed to be related to the increased risk for non‐contact ACL injury [2, 8, 13, 16, 26, 29, 31, 40, 49, 52].

Whereas the anatomical risk factors for ACL injury have been investigated in different patient and athlete populations, most of these past investigations have been relatively small retrospective studies and high‐quality prospective data on this research topic in female athletes is lacking. A better understanding of anatomical risk factors in female athletic populations is needed to inform the development and implementation of targeted prevention strategies. Therefore, the aim of this study was to investigate associations between six selected anatomical variables and non‐contact ACL injuries in female team sport athletes.

## METHODS

### Study design and participants

This study is part of a larger prospective cohort study investigating risk factors for lower limb injuries in team sports (PROFITS Study, 2011–2015) [38]. We invited female basketball, floorball, ice hockey and volleyball teams from the two highest junior leagues and adults' premier league to participate in the study. All female athletes who completed the baseline physical examination were included in this current study.

### Data collection procedures

Athletes entered the study during the preseason (April–May) of 2011, 2012 or 2013, filled out a baseline questionnaire about their background information (e.g., sport, age and injury history) and completed baseline physical examination (height, weight and anatomical tests). Athletes were examined by two physiotherapists and one medical student at the UKK Institute, Tampere, Finland.

#### AP knee laxity

The Knee Laxity Testing Device 1000 (KT‐1000) arthrometer (MEDmetric Corp.) was used to measure AP knee laxity [3, 6]. The athlete was in a relaxed supine position on an examination table. Two trials were performed for both knees, and the maximum value in millimetres for each knee was used for statistical analyses. Based on the International Knee Documentation Committee guidelines, normal knee laxity was defined as a side‐to‐side difference (SSD) of 2 mm or less [19].

#### Genu recurvatum

Knee hyperextension was measured with a standardized goniometer (HiRes goniometer, Baseline® Evaluation Instruments) [15]. The athlete was in a supine position, and a small support was placed under the distal part of the tibia. First, the anterior and posterior parts of the lateral knee joint line were palpated, and a mark was placed at the midpoint of the sagittal plane. Then, the most prominent part of the lateral fibular malleolus and the greater trochanter of the femur were palpated and marked. The axis of the goniometer was placed over the mark on the knee joint line, the arm of the goniometer was lined up with the greater trochanter, and the other arm with the lateral malleolus. The angle was measured to the nearest degree. Plus‐values indicated genu recurvatum.

#### Generalized joint hypermobility

The Beighton score (total of nine points) was used to evaluate generalized joint hypermobility [5, 22]. Because of the analysis based on each leg, the Beighton score was calculated for each half of the body and the forward touchdown was included for both halves of the body (score range: 0–5 points).

#### Hamstrings extensibility

The athlete was lying in supine position on an examination table with a firm surface and lumbar support. The pelvis and the non‐tested leg were stabilized using belts to avoid accessory movements. The hip of the testing leg was fixed at 120° flexion using a belt, and the athlete prevented further hip flexion by pressing with both hands distally on the femur. The ankle and foot were relaxed, and the hip was in neutral rotation, abduction and adduction. Three landmarks were placed on the leg: lateral fibular malleolus, lateral femoral epicondyle and the greater trochanter of femur. The knee was extended passively using a fish scale (Salter Super Samson, Taylor Precision Products, Inc.) with an 8‐kg load. The axis of goniometer was placed over the point of knee joint line and the arms over the lateral malleolus and greater trochanter. The hamstring extensibility was measured as a static range of motion of the knee joint [14].

#### Femoral anteversion

The Craig's test was used to measure the femoral anteversion angle [42]. The athlete was in a relaxed prone position on an examination table, and their pelvis was fixed with a belt. The physiotherapist held around the athlete's distal part of the tibia, passively flexed the knee to 90°, and then rotated the hip passively externally and internally until the most lateral and prominent part of the greater trochanter of the femur was identified by palpation. In this position, the medical student measured the angle between the vertical line and the shaft of the tibia to the nearest degree using a goniometer (Absolute+Axis™ Baseline® Evaluation Instruments) modified with a bubble level to ensure that the stationary arm is held at true vertical. Anteversion is measured as a positive angle and retroversion as a negative angle.

#### Navicular drop

Foot pronation is evaluated by the navicular drop test [43]. The navicular drop was defined as the distance the navicular tuberosity moves in standing, as the subtalar joint is allowed to move from its neutral position to a relaxed position. The athlete stood on the platform, while the most prominent aspect of the navicular bone was marked. To determine navicular height in subtalar joint neutral position, the thumb and forefinger were used to palpate the anterior‐medial and anterior‐lateral head of the talus, respectively, while the athlete rolled the ankle in and out. The subtalar joint neutral position was defined as the position where the medial and lateral aspects of the talar head were equally palpable. From this position, a straight‐edge ruler (positioned perpendicular to the transverse plane) was used to measure the distance from the mark on the navicular to the floor to the nearest millimetre. The athlete was then instructed to relax their foot and evenly distribute weight between the left and right feet. In this relaxed stance, the distance between the mark on the navicular and the floor was again measured. Navicular drop was calculated by subtracting the standing relaxed from the standing subtalar joint neutral navicular height positions.

#### Injury registration

All ACL injuries were recorded through December 2015. During the first 3 study years (May 2011 to April 2014), two study physicians were responsible for collecting the injury data. The physicians contacted the teams (coach, medical staff) once a week to check for possible new injuries. In the final 1.5 study years, the study participants were followed two times (May 2015 and December 2015) with an automatic text message questionnaire. After each reported injury, a study physician or physiotherapist interviewed the injured athlete using a structured injury report form. Only magnetic resonance imaging and/or arthroscopy confirmed non‐contact ACL injuries were included in this study, defined as no direct contact with the involved leg in the injury situation. Recurrent ACL injuries (recurrent ACL injury to the same knee as index injury) were excluded from the analysis. All participants diagnosed with an ACL rupture subsequently underwent ACL reconstruction.

### Statistical analysis

Statistical analyses were performed with Statistical Product and Service Solutions (SPSS) (Version 29.0; SPSS, Inc.). Study participants were divided into two groups: (1) ACL injury group, which sustained a noncontact ACL injury during the follow‐up period, and (2) no ACL injury group. Normal distribution was assessed prior to analysis. Independent samples *t*‐tests and a chi‐square test were used to compare the two groups. The *t*‐test was performed using each leg as a unit of analysis, and the chi‐square test was performed using an athlete as a unit of analysis. The main analysis was conducted using univariate logistic regression. Statistical significance was set at *p* < 0.05. Based on Bahr and Holme [4], in order to detect moderate to strong associations between risk factors and injury, 20–50 injury cases were needed for analysis.

## RESULTS

Complete baseline and follow‐up data were obtained from 287 study participants (Table 1). Eight participants had a history of ACL injury at baseline (floorball: 5; basketball: 2; volleyball: 1), and their injured legs were excluded. A total of 566 legs were included in the analysis. The mean follow‐up time was 203 weeks (standard deviation [SD]: ±39). During the follow‐up, 29 ACL injuries occurred in the included legs. Three participants experienced recurrent ACL injury to the same leg during the study, but only their initial non‐contact injury was included in the analysis. Additionally, three direct contact injuries were excluded. In total, 23 non‐contact ACL injuries were analyzed (Table 1).

**Table 1:** Athlete characteristics and ACL injuries by sport.

	Basketball (*n* = 106)	Floorball (*n* = 105)	Ice hockey (*n* = 58)	Volleyball (*n* = 18)	ALL (*n* = 287)
Athlete characteristics^a^					
Age at baseline (years)	14.8 ± 2.0	18.7 ± 4.7	19.2 ± 3.6	16.9 ± 0.7	17.2 ± 3.9
Height (cm)	168.7 ± 6.8	166.5 ± 5.7	165.4 ± 6.3	175.8 ± 7.0	167.7 ± 6.8
Body mass (kg)	61.4 ± 9.8	61.9 ± 2.5	65.5 ± 6.6	67.9 ± 7.1	62.8 ± 8.4
ACL injury during follow‐up^b^	6	20	1	2	29
Recurrent injury^c^	1	2	0	0	3
Direct contact injury^d^	0	2	1	0	3
Non‐contact injury	5	16	0	2	23

Abbreviations: ACL, anterior cruciate ligament; SD, standard deviation.

^a^ Results given as mean ± SD.

^b^ Results given as number of ACL injuries.

^c^ Recurrent ACL injury to the same knee during the follow‐up.

^d^ Direct contact with the injured limb at the time of injury.

The mean age at the time of non‐contact ACL injury was 18.3 years (SD: ±3.0). The median time to non‐contact ACL injury from baseline was 14 months (range: 3–51 months). Nine players sustained an injury within 10 months of the baseline, six players sustained an injury between 13 and 19 months, and the remaining eight players sustained an injury after 26 months. Baseline physical examination variables were not significantly different between the groups with and without a non‐contact ACL injury during the follow‐up (Tables 2 and 3). Univariate logistic regression did not reveal any significant findings (Table 4).

**Table 2:** Comparison of baseline physical examination variables between the groups.

	ACL injury (*n* = 23)	No ACL injury (*n* = 543)	*p*‐Value
Age at baseline (years)	16.7 ± 2.8	17.2 ± 4.0	0.52
Height (cm)	168.8 ± 7.4	167.6 ± 6.7	0.42
Body mass (kg)	62.9 ± 7.8	62.8 ± 8.4	0.96
KT‐1000 (mm)	6.4 ± 2.1	6.7 ± 2.0	0.56
Genu recurvatum (°)	6.6 ± 3.2	6.0 ± 4.0	0.46
Generalized hypermobility^a^	1.7 ± 1.2	1.5 ± 1.1	0.34
Femoral anterversion (°)	9.9 ± 6.3	8.3 ± 6.2	0.24
Hamstring extensibility (°)	146.0 ± 15.4	147.4 ± 14.8	0.65
Navicular drop (mm)	5.4 ± 2.9	6.3 ± 3.7	0.26

*Note*: Results given as mean ± SD. *p*‐Values shown refer to the *t*‐test between legs with and without a non‐contact ACL injury.

Abbreviations: ACL, anterior cruciate ligament; KT‐1000, Knee Laxity Testing Device 1000; SD, standard deviation.

a Generalized hypermobility for each half of the body (score range: 0–5).

**Table 3:** SSDs in anterior‐posterior knee laxity in female athletes with and without an ACL injury during the follow‐up.

	ACL injury (*n* = 20)	No ACL injury (*n* = 259)	*p*‐Value
SSD ≤ 2 mm, *N* (%)	16 (80)	228 (88)	
			0.30
SSD > 2 mm, *N* (%)	4 (20)	31 (12)	

*Note*: Results are given as number (percentage) of injured athletes. *p*‐Value refers to the chi‐square test. Athletes with a history of ACL injury (*n* = 8) were excluded from the analysis.

Abbreviations: ACL, anterior cruciate ligament; SSD, side‐to‐side difference.

**Table 4:** Univariate logistic regression for the anatomic variables.

	OR	95% CI	*p*‐Value
KT‐1000max	0.97	0.81–1.16	0.72
Side‐to‐side difference	0.66	0.25–1.73	0.40
Genu recurvatum	1.02	0.93–1.12	0.64
Generalized hypermobility	1.10	0.80–1.51	0.55
Femoral anterversion	1.02	0.96–1.09	0.47
Hamstring extensibility	1.00	0.97–1.02	0.78
Navicular drop	0.97	0.87–1.07	0.50

Abbreviations: CI, confidence interval; KT‐1000, Knee Laxity Testing Device 1000; OR, odds ratio.

## DISCUSSION

This study investigated associations between previously proposed anatomical risk factors and non‐contact ACL injury in female team sport athletes. Several previous studies have suggested that AP laxity of the knee is associated with the risk for ACL injury in female athletes [13, 31, 46, 47, 52]. However, contradictory results have also been published [33, 49]. Four of these earlier studies used prospective data collection, others were small retrospective case–control studies. Vacek et al. [47] conducted a prospective cohort study with a nested case–control analysis among high school and collegiate athletes. Seventy female athletes sustained an ACL injury during the 4‐year follow‐up. After the injury but before surgery, injured athletes and their matched controls completed a physical examination. The results showed that greater AP knee laxity measured from the uninjured knee in athletes who sustained an ACL injury, a higher body mass index (BMI), and a parental history of ACL injury were associated with an increased risk of ACL injury in female athletes. In another nested case–control study [31], female basketball and soccer athletes were prospectively tested for laxity measures before their ACL injury. Comparison between injured (*n* = 19) and matched controls (*n* = 76) revealed that SSDs in AP laxity and increased genu recurvatum increased the risk of ACL injury. In this current study, AP knee laxity was not found to be a risk factor, which supports other prospective cohort studies in female athletes [33, 49]. However, the prevalence of SSD >2 mm was 20% in the ACL injury group and 12% in the no ACL injury group. Hence, it could be worth investigating AP laxity and ACL injury risk in future studies with larger sample sizes and/or longer follow‐up times.

In contrast to previous studies [26, 29, 31, 40, 49], association between genu recurvatum and ACL injury risk was not found in our study. A prospective cohort study [49] in female team sport athletes found a statistically significant difference between the ACL‐injured (*n* = 11) and uninjured (*n* = 520) groups in baseline genu recurvatum, with injured athletes having greater knee extension. Also, the nested case–control study by Myer et al. [31] suggested that increased genu recurvatum is associated with the risk of ACL injury. Other previous studies were retrospective case–control studies with possible biases.

In the current study, generalized joint hypermobility was not a risk factor for non‐contact ACL injury. This finding also stands in contrast to previous studies [26, 40, 46, 48]; however, of these, only one was a prospective cohort study. In that study, with a 4‐year follow‐up, Uhorchak et al. [46] examined anatomical risk factors for ACL injuries in military population. Their results suggested that generalized joint hypermobility and a higher than average BMI were statistically significant risk factors among female participants, with 8 female athletes sustaining an ACL injury and 112 remaining injury‐free. However, the results of this study have limited generalizability because of a different risk profile (extrinsic and intrinsic risk factors) in the military population compared to female team sports. The results of this current study align with those of another prospective cohort study [33] involving youth female basketball and handball athletes (*n* = 317; 27 non‐contact ACL injuries), which also found no association between generalized joint hypermobility and ACL injury.

Femoral anteversion has been suggested as a potential anatomical factor that may contribute to ACL injury risk [2, 13]. It has been suggested that increased femoral anteversion may alter lower limb alignment and mechanics, leading to increased stress on the ACL during dynamic movements [23]. A retrospective case–control study by Alpay et al. [2] found significantly higher femoral anteversion angles in the ACL‐deficient group (*n* = 28) than in the control group (*n* = 28); however, the majority of participants in both groups were males. Another case–control study by Davey et al. [13] has suggested that increased femoral anteversion is associated with the risk of secondary ACL injury on the contralateral knee in female high school athletes. Our study, as well as a recent systematic review, did not support these findings [18].

Previous biomechanical studies have indicated that the increased risk of ACL injuries in female athletes might be associated with inadequate hamstring stiffness and increased hamstring extensibility [7, 8]. In theory, higher hamstring stiffness can more effectively reduce ACL loading, such as anterior tibial shear force, during landing compared to lower hamstring stiffness. This increased hamstring stiffness is also associated with reduced hamstring extensibility [7, 8]. The greater resistance to knee extension resulting from higher hamstring stiffness and lower extensibility may contribute to improved landing biomechanics, such as increased knee flexion during landing, thereby reducing the risk of ACL injuries. This was investigated by Boden et al. [10], who reported that participants with ACL injuries displayed greater hamstring extensibility than uninjured athletes. In this current study, we did not measure hamstring stiffness, but we measured hamstring extensibility. Our findings did not support earlier suggestions related to the association between hamstring extensibility and ACL injury risk.

There have been a few studies suggesting that greater navicular drop may contribute to ACL loading and hence injury risk [16, 29, 52]. One proposed mechanism is that excessive navicular drop may lead to altered lower limb biomechanics [16], such as increased knee valgus, which could potentially increase stress on the ACL during cutting, pivoting and landing activities. However, studies that have found a significant association between navicular drop and ACL injury risk have been small retrospective case–control studies, whereas prospective cohort studies [33, 49], including our current study, did not support these findings.

The present study has many strengths compared with previous risk factors studies, such as a larger sample size and a relatively long follow‐up period. In contrast to previous studies, all participants of this current study were youth and adult female athletes from the highest league levels, making our cohort more homogenous compared to earlier studies. The current study also has certain limitations that should be considered. First, the anatomical variables measured at baseline may have changed over the long follow‐up period, particularly in adolescent participants. However, 96% of our participants had experienced menarche, meaning that they had passed their peak height velocity prior to the study onset. Second, participants' menstrual cycle phase was not determined when they completed the baseline physical examination. The menstrual cycle and hormonal effects can affect ligament laxity and hamstring extensibility [44]. Third, because the physical examination included manual clinical testing, some degree of measurement error is expected. To minimize these errors, we used the same two experienced physiotherapists and a medical student throughout the testing years. The physiotherapists conducted all manual tests, while the medical student assisted with tasks such as placing the fixation belts and recording the test results. Physiotherapist 1 was responsible for measuring the navicular drop, while Physiotherapist 2 handled the KT‐1000, Beighton score, genu recurvatum, hip anteversion and hamstring extensibility. Intra‐rater reliability has been found to be satisfactory for the genu recurvatum test [39], acceptable for the KT‐1000 assessment [41], moderate to good for the navicular drop test [50], and highly reliable for both the Beighton [9] and Craig's tests [11]. However, recent findings have suggested that the Craig's test may not be a valid clinical assessment of true femoral version [12, 20].

The final limitation of our study was the limited number of ACL injuries. This study was part of a larger cohort study investigating risk factors for lower limb injuries in female and male team sport athletes, and the power estimations were made for the larger study, not for this current study. The present study, which included 23 non‐contact ACL injuries in 287 athletes, was powered to detect moderate to strong associations between risk factors and outcomes. Detecting small to moderate risk associations would require approximately 200 injury cases for analysis [4]. As ACL injuries are relatively rare, prospective cohort studies require either larger sample sizes or longer follow‐up periods for statistically robust results.

## CONCLUSIONS

Anatomical characteristics, including AP knee laxity, knee hyperextension, generalized hypermobility, femoral anteversion, hamstring extensibility and navicular drop, do not play important roles in non contact ACL injury risk in adolescent and adult female team sport athletes. However, the number of participants with an ACL injury was limited in this prospective cohort study, and therefore, it is important to be cautious when interpreting these findings. Minor risk associations may not have been detected in the current study. We recommend that future longitudinal studies on ACL risk factors include a larger number of study participants and a longer follow‐up time.

## AUTHOR CONTRIBUTIONS

Kati Pasanen, Jari Parkkari, Tommi Vasankari, Grethe Myklebust and Tron Krosshaug contributed to the development of the study proposal. Kati Pasanen, Jari Parkkari and Tommi Vasankari contributed to the funding acquisition. Kati Pasanen and Mari Leppänen contributed to data collection, data entry and data cleaning. Kati Pasanen, Arttu Seppänen and Kari Tokola contributed to the data analysis and interpretation of study results. Kati Pasanen led all aspects of the study project. Kati Pasanen wrote the first draft of the manuscript. All authors critically reviewed and edited the manuscript before submission.

## CONFLICT OF INTEREST STATEMENT

The authors declare no conflicts of interest.

## ETHICS STATEMENT

This study was approved by the Ethics Committee of Pirkanmaa Hospital District (ethics approval number R10169). Informed consent was obtained from all individual participants included in the study (including parental consent for athletes <18 years).

## Data Availability

The data collected and analyzed during the current study are not publicly available. Access to the data is restricted to members of the research group only.
